# Avian Egg Odour Encodes Information on Embryo Sex, Fertility and Development

**DOI:** 10.1371/journal.pone.0116345

**Published:** 2015-01-28

**Authors:** Ben Webster, William Hayes, Thomas W. Pike

**Affiliations:** School of Life Sciences, University of Lincoln, Lincoln, United Kingdom; University of Milan, ITALY

## Abstract

Avian chemical communication is a rapidly emerging field, but has been hampered by a critical lack of information on volatile chemicals that communicate ecologically relevant information (semiochemicals). A possible, but as yet unexplored, function of olfaction and chemical communication in birds is in parent-embryo and embryo-embryo communication. Communication between parents and developing embryos may act to mediate parental behaviour, while communication between embryos can control the synchronicity of hatching. Embryonic vocalisations and vibrations have been implicated as a means of communication during the later stages of development but in the early stages, before embryos are capable of independent movement and vocalisation, this is not possible. Here we show that volatiles emitted from developing eggs of Japanese quail (*Coturnix japonica*) convey information on egg fertility, along with the sex and developmental status of the embryo. Specifically, egg volatiles changed over the course of incubation, differed between fertile and infertile eggs, and were predictive of embryo sex as early as day 1 of incubation. Egg odours therefore have the potential to facilitate parent-embryo and embryo-embryo interactions by allowing the assessment of key measures of embryonic development long before this is possible through other modalities. It also opens up the intriguing possibility that parents may be able to glean further relevant information from egg volatiles, such as the health, viability and heritage of embryos. By determining information conveyed by egg-derived volatiles, we hope to stimulate further investigation into the ecological role of egg odours.

## Introduction

The eggs of many species of fish and insects emit volatile compounds that act, incidentally, as cues to predators and parasites as to their location [1–4]. Birds’ eggs also produce odorous compounds during incubation [5] although their ecological significance has been almost completely unexplored; not least because until recently the sense of smell in birds has been largely underappreciated [6–11]. One possible role for egg odours may be as a component of parent-embryo and embryo-embryo interactions. It is well-established that embryo-parent communication occurs late on during incubation, when the embryo first begins to vocalise within the shell [12]. This is used to communicate thermal status [13,14] and may also be used to advertise developmental status, synchronise hatching, solicit care and discourage nest abandonment [15–17]. However, because the embryonic vocalisation system does not develop until a few days prior to hatching [12], this would not be a possible means of embryo-parent communication during the majority of incubation. Instead, chemical cues released through the porous egg shell may allow parents to assess the status of developing embryos. For example, nitric oxide (NO) produced by developing embryos has been suggested to play a role in mediating brood-patch development, although this has been viewed as a passive process in which released NO directly stimulates development [18] and not as an active process in which egg odours mediate adaptive behavioural changes in incubating parents.

Embryo-embryo communication can facilitate the timing of hatching, which is important for early performance in many bird species [19]. In Japanese quail (*Coturnix japonica*), for example, placement of eggs at different stages of development in close proximity can act to advance or retard development so that hatching occurs synchronously, and both vocalisations and vibrations emanating from within adjacent eggs have been implicated as possible mechanisms [20]. However, as discussed above such cues are only useful during the latter stages of incubation, and no study has yet attempted to confirm or refute the possibility that the exchange of chemical cues between eggs may play a role in embryo-embryo communication.

Before the ecological role of egg odours can be investigated, however, the information conveyed by egg-derived volatiles must first be determined. Avian chemical ecology is a rapidly emerging field but is hampered by a critical shortage of information on bird semiochemicals [7] and no study has yet determined what information, if any, is conveyed in egg odours. In this paper, we look at odours produced by Japanese quail eggs during incubation, and specifically test whether egg volatiles reflect egg fertility, embryonic development, and embryo sex; information which is potentially valuable in both parent-embryo and embryo-embryo communication. At the beginning of incubation the embryo is poorly developed, with no known differences between male and female embryos [21] and no reason to expect compositional differences between eggs containing fertile or infertile embryos. All eggs should therefore have similar odours. However, as incubation progresses, it is expected that volatiles produced as a direct result of embryonic metabolism will result in increasingly divergent volatile composition between fertile and infertile eggs, while sex differences in embryonic growth rate, metabolism or physiology [22–25], will result in emergent sex differences. We therefore make two specific predictions: (1) there will be an interaction between fertility and developmental stage, with egg volatile composition initially not differing between fertile and infertile eggs, but diverging later in development; and (2) there will be an interaction between embryo sex and developmental stage, with no sex differences during the early stages of incubation, but differences between eggs containing male and female embryos emerging during later stages.

## Materials and Methods

### (a) Egg incubation

Freshly-laid Japanese quail eggs were obtained from a commercial supplier (Paslow Common Farm, Essex, UK) and stored in a refrigerator at 4°C for no more than 5 days until use. Immediately prior to incubation, eggs were gently wiped with ethanol to remove any residue before being quickly rinsed with water and dried using a paper towel. Eggs were then transferred to an incubator (Octagon 20 Advance Incubator with autoturn cradle, Brinsea Products Ltd, Sandford, UK) maintained at 37.5°C and 60% humidity, and incubated for 8 days (out of a total incubation period of ~17 days). This reflects the developmental period we are most interested in, prior to the onset of auditory and vibrational sensitivity during the second half of incubation [26,27].

### (b) Volatile collection

Volatile collection was performed on day 1 and day 8 of incubation, as follows. A single egg was transferred to a glass jar (50 mm high, 34 mm diameter) sealed with aluminium foil and placed on a heating block to maintain the temperature at 37.5°C. The egg was left for 20 min to allow volatiles to equilibrate with the headspace of the jar, after which a 60μm PDMS/DVB Stableflex 24 gauge solid phase microextraction (SPME) fibre with holder (Supelco, Bellefonte, PA, USA) was inserted through the aluminium foil for 20 min. Eggs were returned to the incubator immediately following volatile collection. Volatiles were collected from empty glass jars, which were otherwise treated in exactly the same way, for comparison. After day 8, eggs were opened to check for the presence of developing embryos. In total, 65 eggs were incubated and subject to volatile collection, 50 of which had a developed embryo on day 8 and 15 of which had no visible embryo and so were deemed infertile. Incubation and volatile collection was carried out over two separate occasions, with approximately half the eggs present together in the same incubator on each occasion.

### (c) Chemical analysis

Chemical identification was achieved by coupled gas chromatography-mass spectrometry (GCMS). Immediately after volatile collection, the SPME fibre was transferred to the injector port of a Shimadzu GCMS-QP2010S fitted with an HP-1MS Ultra Inert column (30m x 0.25mm, 0.25um film thickness). The injector port was at 250°C in splitless mode and fitted with an SPME liner. Oven temperature was maintained at 40°C for 1 min, then programmed at 10°C min^−1^ until 240°C and held for 15 min. The carrier gas was helium at 1 ml min^−1^. The transfer line temperature was 240°C and ion source was at 200°C. Ionization was by electron impact at 70eV. Peaks that were consistently present in greater amounts than in empty glass jar controls were identified by comparison of spectra with those of a database (NIST 05) and confirmed by comparison of mass spectra and retention times with those of commercially available authentic standards of each putative compound.

Quantification was achieved using single ion counts of identified peaks, selecting ions that were not present in neighbouring peaks on the chromatogram. Calibration curves were constructed for each compound by injecting authentic standards into a conventional injection port liner at a range of known concentrations in acetonitrile. In total 24 volatile compounds were detected (Table 1, S1 and S2 Tables), three of which could not be identified (denoted unidentified 1–3): they had Kovats retention indices of 988, 1087, and 1128, respectively, and their dominant ions were m/z 81, 110 and 79 (unidentified 1); 67, 95 and 124 (unidentified 2); and 41, 55 and 97 (unidentified 3). Since we could not determine concentrations of these compounds, for analysis we used mean single ion counts for these unidentified compounds (1, m/z 110; 2, m/z 124; and 3, m/z 97) and 1,3-diphenyl propane (m/z 92), for which no authentic standard was commercially available.

**Table 1:** Correlations of canonical axes of principle coordinates (CAP) axes with compounds identified in egg odour.

	Fertility		Sex	
Compound	CAP axis 1	CAP axis 2	CAP axis 1	CAP axis 2
1-butanol	0.2176	-0.0377	-0.4865	-0.0605
dimethyl disulfide	0.3795	0.1124	-0.3786	-0.0355
methyl benzene	0.6733	0.1113	-0.7812	-0.2709
hexanal	0.0567	0.1195	-0.0774	-0.3352
phenylethene	0.7393	0.043	-0.8024	-0.2968
heptanal	-0.161	-0.1128	0.2805	-0.2593
benzaldehyde	0.3594	-0.1113	-0.3292	-0.6876
dimethyl trisulfide	0.1287	-0.0201	-0.0233	-0.0408
phenol	0.2887	0.0595	-0.2189	-0.5581
2-(2-ethoxyethoxy)ethanol	0.0166	0.04	0.0172	-0.2789
unidentified 1	-0.3464	-0.1956	0.5767	-0.1994
2-ethyl-1-hexanol	0.1305	-0.0021	-0.1342	-0.5011
5-isopropenyl-1-methyl-1-cyclohexene	0.8991	0.0891	-0.8486	-0.223
acetophenone	0.3348	0.0856	-0.3306	-0.5949
2-nonanone	-0.046	0.144	0.0919	-0.616
unidentified 2	-0.5174	-0.1318	0.5943	-0.1599
unidentified 3	0.1298	0.0505	-0.1135	-0.331
2-decanone	-0.2261	0.0711	0.2889	-0.625
2-isopropylphenol	0.0479	0.0468	-0.0533	-0.4098
benzothiazole	0.2759	0.1034	-0.324	-0.4438
2-undecanone	-0.065	-0.0689	0.2334	-0.525
1,3-diacetylbenzene	-0.467	-0.039	0.5657	-0.2151
diethyl phthalate	0.0611	0.0708	0.0829	-0.2881
1,3-diphenyl propane	0.149	0.0198	-0.0619	-0.3059

**Figure 1:**
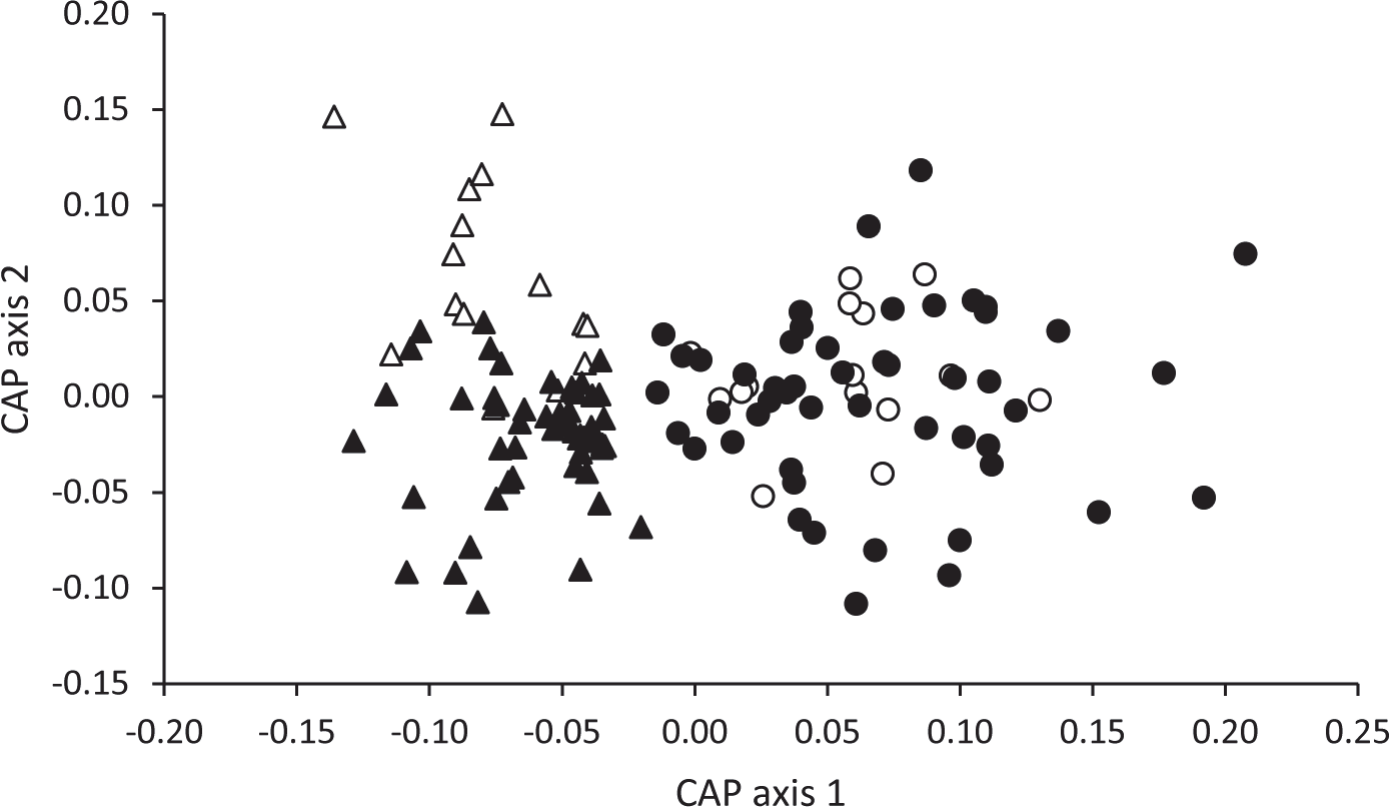
Temporal variation in the odour profiles of fertile and infertile eggs. Canonical analysis of principal coordinates (CAP) showing separation of the multivariate odour composition of fertile (black data points) and infertile (white data points) eggs on day 1 (circles) and day 8 (triangles) of incubation.

**Figure 2:**
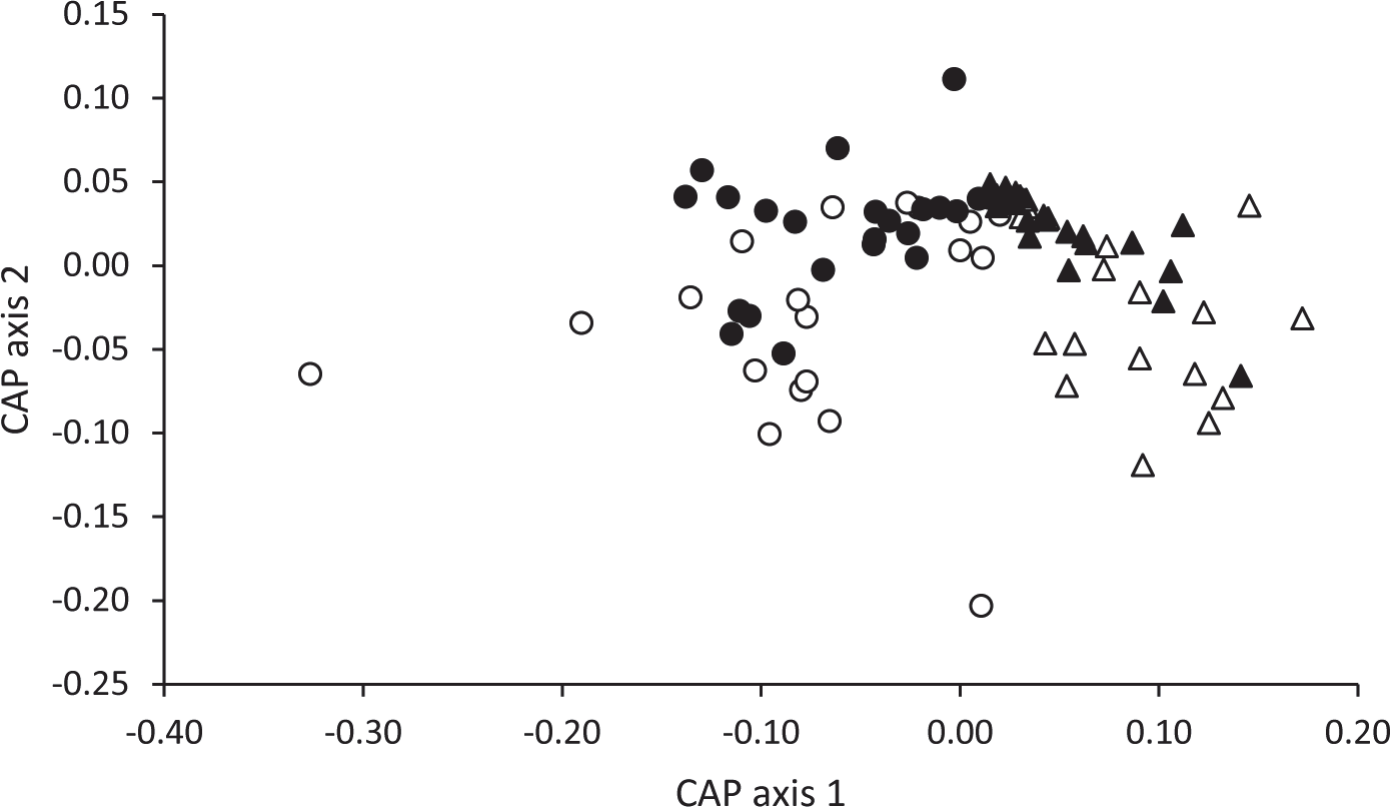
Temporal variation in the odour profiles of eggs containing male and female embryos. Canonical analysis of principal coordinates (CAP) showing separation of the multivariate odour composition of eggs containing male (black data points) and female (white data points) eggs on day 1 (circles) and day 8 (triangles) of incubation.

For the analysis of the fertility data, CAP axis 1 corresponds to separation between day 1 and day 8 eggs, and CAP axis 2 corresponds to separation between fertile and infertile eggs (see also Fig. 1). For the analysis of the sex data, CAP axis 1 corresponds to separation between day 1 and day 8 eggs, and CAP axis 2 corresponds to separation between eggs containing male and female embryos (see also Fig. 2). Absolute correlation coefficients >0.5 are shown in bold.

### (d) Molecular sexing

Following collection of volatiles on day 8, a random sample of developed embryos (n = 42) were subject to molecular sexing, blind and in a random order. Embryos were weighed (± 0.01 g) and genomic DNA was extracted from approximately 0.1 g embryonic tissue using the DNeasy Blood & Tissue Kit (Qiagen, Valencia, CA). Samples were sexed using PCR amplification of part of the sex-linked *CHD1* genes, *CHD1W* and *CHD1Z*, which map to the avian W and Z chromosomes, respectively, using primers 2718R and 2550F [28]. PCR products were separated on 2% agarose gels and visualised with ethidium bromide staining. Females were characterized by displaying a *CHD1W*-specific fragment (1.2 kb in size) plus a shorter *CHD1Z*-specific fragment (0.7 kb), while males showed only the shorter Z-fragment.

### (e) Statistical analysis

Quantities of each detected compound were standardised by dividing by the highest concentration of that compound collected. Four compounds that could not be quantified (unidentified 1, 2 and 3 and 1,3-diphenyl propane) were assumed to have a linear relationship between single ion count and quantity and standardised in the same way as for identified compounds. Since we had no a priori knowledge of which compounds are most important or most readily detected or discriminated, this transformation ensured that all compounds were treated equally in the analysis and there was no bias towards particularly abundant compounds.

To test whether variation in the composition of volatiles (i.e., odour profiles) was explained by an interaction between fertility and day of incubation, as predicted, we performed a permutational (non-parametric) multivariate analysis of variance (MANOVA) [29], using the ‘adonis’ function in the ‘vegan’ package [30] for R (version 2.15.2), with the interaction between fertility (fertile or infertile) and day of incubation (day 1 or day 8) as a fixed effect and permutations constrained by egg identity to account for measurements being taken from the same eggs on day 1 and day 8 of incubation. Post-hoc pairwise comparisons were conducted using canonical analyses [21,22], which include a permutation test of differences in odour composition between subgroups, and outputs the largest root test statistic, δ_1_^2^. To test our second prediction, that variation in the odour profiles of fertile eggs could be explained by an interaction between embryo sex and stage of development, we performed a similar non-parametric MANOVA exploring the interaction between embryo sex and day of incubation. This general approach has been successfully used elsewhere to test for differences in avian odour composition [31].

Odour composition data were visualised using canonical analysis of principal coordinates (CAP) [32,33], a multivariate ordination technique. Pearson’s correlations between canonical axes and standardised abundance data for each compound were used to indicate the relative contribution of each compound to any separation observed [31–33], with greater absolute values of the correlation coefficient denoting a greater contribution of that compound to separation between groups. Note that these correlations should not be interpreted in a causative way [31], and so we do not attempt to assign statistical significance to them (*cf*. [31]); instead, we simply note which compounds make a relatively large contribution to the observed separation (arbitrarily defined as those with an absolute correlation coefficient >0.5).

### (f) Ethical statement

This study was approved by the University of Lincoln Ethical Review Committee, and was conducted in strict accordance with the laws of the UK.

## Results

### (a) Embryo fertility and development

There was a significant interaction between fertility and day of incubation in explaining variation in odour composition (F_3,126_ = 4.49, P < 0.001). All eggs exhibited a change in odour composition between day 1 and day 8 (fertile eggs: δ_1_^2^ = 0.79, P < 0.001; infertile eggs: δ_1_^2^ = 0.85, P < 0.001); however, while there was no significant difference in volatile composition between fertile and infertile eggs on day 1 (δ_1_^2^ = 0.15, P = 0.738), by day 8 the difference was highly significant (δ_1_^2^ = 0.86, P < 0.001) (Fig. 1).

The greatest contributions to separation along CAP axis 1 (Fig. 1), which corresponds to separation according to day of incubation, were made by methyl benzene, phenylethene, 5-isopropenyl-1-methyl-1-cyclohexene (which were more abundant on day 1 than day 8; S1 Table) and unidentified 2 (which was most abundant on day 8; S1 Table). No single compound contributed substantially to separation of volatile profiles from fertile and infertile eggs (along CAP axis 2 in Fig. 1) (Table 1).

### (b) Embryo sex and development

Variation in odour composition was significantly explained by the interaction between embryo sex and day of incubation (F_3,80_ = 4.76, P < 0.001). There was a significant change in volatile composition between days 1 and 8 for eggs containing both male (δ_1_^2^ = 0.92, P < 0.001) and female (δ_1_^2^ = 0.57, P < 0.001) embryos, and, surprisingly, there were differences in odour profiles between eggs containing male and female embryos on both day 1 (δ_1_^2^ = 0.63, P = 0.042) and day 8 (δ_1_^2^ = 0.57, P = 0.014) (Fig. 2). There was no difference in weight between male and female embryos on day 8 (two-sample t-test: t_38_ = −0.01, P = 0.995).

For day of incubation (separation along CAP axis 1 in Fig. 2), the greatest contributions were made by methyl benzene, phenylethene, 5-isopropenyl-1-methyl-1-cyclohexene and unidentified 2, as in the fertility analysis, with the addition of unidentified 1 and 1,3-diacetylbenzene (both most abundant on day 8; S1 Table). The compounds contributing the most to separation of volatile profiles from male and female eggs (along CAP axis 2 in Fig. 2) were benzaldehyde, phenol, acetophenone, 2-nonanone, 2-decanone and 2-undecanone (Table 1), which were all more abundant in eggs containing female embryos (S1 Table).

## Discussion

The results of this study show that both the fertility of an egg and the day of incubation interactively affect the composition of volatiles emitted during incubation, and, for fertile eggs, volatile composition is predictive of embryo sex, not only on day 8 as hypothesised but also on day 1 of incubation. To our knowledge, this is the first study to characterise the odour profiles of bird eggs containing developing embryos. As such, many of those volatiles identified are not widely reported in the literature and add to our limited knowledge of potential bird semiochemicals [7].

The lack of difference observed between the odour profiles of fertile and infertile eggs on day 1 was not unexpected since at this stage embryonic development has barely begun and there would be no reason to predict differences in egg composition. By day 8, however, a significant difference in volatile composition had developed, presumably driven by a combination of embryonic metabolism and utilisation of egg components that were absent from infertile eggs, possibly augmented by biochemical and microbial changes in infertile eggs [34]. Birds invest considerable time and energy in the production and incubation of eggs and being able to detect fertile eggs within a clutch could be advantageous, for example to facilitate decisions on whether or not to abandon the nest in the event of increased predation or reduced availability of food resources; although whether olfaction could be used to mediate nest abandonment decisions is not known and would make an interesting area of future research.

The finding that the volatile composition differed between eggs containing male and female embryos, not only on day 8—by which time sex differences in embryonic growth rate or selective utilisation of egg components [35] may have contributed to the observed differences—but also on day 1 was extremely unexpected. It is unlikely that action by the embryos themselves could have contributed to this difference (although this cannot be ruled out entirely), and so we suggest that the observed differences in odour composition may have been driven by differential maternal allocation of resources to male and female eggs, resulting in differential emission of volatiles. For example, female birds are known to allocate various yolk components, including hormones [36], antibodies [37] and possibly antioxidants and vitamins [38], to eggs in a sex-specific manner, although it has never been investigated whether these compounds could contribute to the odour of avian eggs. However, it is noteworthy that one of the volatile ketones which differed between eggs containing male and female embryos (2-undecanone), has previously been identified as a hormone-linked constituent of avian odour [39,40]. Regardless of the underlying mechanism, the implications of sex differences in the volatiles from avian eggs are considerable. In particular it may provide a mechanism by which parents can selectively allocate incubation effort to eggs of one sex over the other, perhaps to differentially enhance or retard growth in species with hatching asynchrony [23,41], and possibly to form the basis for a post-ovulatory means of sex ratio manipulation [42]. More pragmatically, the poultry industry would benefit from the ability to detect egg sex at an early developmental stage [43].

Some compounds showed a marked decrease in emission from day 1 to day 8 of incubation. Chicken (*Gallus gallus*) eggs are capable of absorbing extraneous odours [44] and it is possible that some volatiles may have been absorbed through the porous shell during or after laying and then re-emitted, at decreasing rates as specific volatiles are expended, over time. A similar phenomenon has been observed in plants that can absorb and re-emit volatiles produced by neighbouring plants [45]. This could explain the emission patterns of compounds such as methylbenzene and phenylethene, which are not commonly associated with biological activity. However, several compounds (e.g. 1,3-diacetyl benzene, heptanal and unidentified 1 and 2 in S1 Table) were produced in greater quantities by day 8 eggs compared to day 1 eggs showing that differences in odour over time are not solely due to gradual depletion of absorbed volatiles. If eggs are capable of absorbing odours from the nest or from the incubating parent, this raises the possibility that absorbed odours may facilitate egg recognition. Japanese quail can detect visual differences between host and foreign eggs, although the use of visual cues alone may not be sufficient when differences in maculation patterns and shape are minor [46]. Given the accumulating evidence that birds can recognise the odour of their nests [8,9,47], odour cues may facilitate egg recognition in such situations which could possibly be aided by absorption of nest odours.

Chemical communication between animals has largely focussed on insects and mammals. The lack of such studies on birds is probably a result of an under-appreciation of the avian olfactory sense, despite an increasing number of studies showing that olfaction is a well-developed and ecologically important trait in many bird species [6,7]. This is the first study to characterise the volatile chemicals given off by developing bird eggs and show that these volatiles convey ecologically relevant information on the developmental status of the embryo. Whether or not these volatiles play a role in mediating parental incubation behaviour or embryo-embryo communication remains to be determined, however, and future work should address this, for example by employing artificial eggs emitting volatile blends based on those identified. Egg odours may also provide information on a range of other factors relevant to egg development long before this information is available via other sensory modalities. These include embryonic health (for example volatile markers of oxidative stress caused by the breakdown of lipids by rapidly developing embryos [48], or odours resulting from microbial infection), growth rate, egg temperature, and the recognition of eggs from inter- and intra-specific brood parasites. For instance, hexanal is an important volatile decomposition product of hydroperoxides formed from *n*-6 polyunsaturated lipids [49], which are present in the yolk of quail eggs [50], and so may indicate embryonic susceptibility to lipid peroxidation. Odours may also be used to solicit parental care and allow assessment of maternal quality [51], although the behavioural responses of parents to variation in egg odour remains to be established. These results therefore open up a host of interesting questions on the possible role of egg semiochemicals, and could pave the way for further advances in avian chemical ecology.

## Supporting Information

S1 Table Egg volatiles as a function of developmental stage and fertility. List of compounds and mean (± SE) quantities (ng) collected over 20 min from fertile and infertile Japanese quail eggs on day 1 and day 8 of incubation.
(PDF)

S2 Table Egg volatiles as a function of developmental stage and embryo sex. List of compounds and mean (± SE) quantities (ng) collected over 20 min from Japanese quail eggs containing male and female embryos on day 1 and day 8 of incubation.
(PDF)
