# Supplementary material for: Avian Egg Odour Encodes Information on Embryo Sex, Fertility and Development
Source: PLoS One. 2015 Jan 28;10(1):e0116345. doi: 10.1371/journal.pone.0116345 (PMC4309571; doi:10.1371/journal.pone.0116345)
Supplement: S2 Table — List of compounds and mean (± SE) quantities (ng) collected over 20 min from Japanese quail eggs containing male and female embryos on day 1 and day 8 of incubation. (PDF) [file pone.0116345.s002.pdf]

Table S2. Egg volatiles as a function of developmental stage and embryo sex. List of compounds and mean (± SE) quantities (ng) collected over 20 min from Japanese quail eggs containing male and female embryos on day 1 and day 8 of incubation.

	**Day 1**		**Day 8**	
**Compound**	**Male**	**Female**	**Male**	**Female**
1-butanol	0.985 (0.294)	1.135 (0.422)	0.652 (0.129)	0.400 (0.093)
dimethyl disulfide	0.257 (0.076)	0.170 (0.039)	0.041 (0.022)	0.098 (0.026)
methyl benzene	0.364 (0.053)	0.485 (0.140)	0.047 (0.006)	0.030 (0.005)
hexanal	0.711 (0.127)	0.859 (0.173)	0.502 (0.207)	0.772 (0.142)
phenylethene	0.895 (0.138)	1.054 (0.225)	0.028 (0.004)	0.027 (0.005)
heptanal	0.289 (0.073)	0.332 (0.078)	0.385 (0.178)	0.728 (0.178)
benzaldehyde	1.412 (0.270)	1.925 (0.305)	0.540 (0.126)	1.537 (0.283)
dimethyl trisulfide	0.069 (0.028)	0.042 (0.011)	0.022 (0.015)	0.061 (0.016)
phenol	0.589 (0.140)	1.050 (0.253)	0.318 (0.073)	0.599 (0.112)
2-(2-ethoxyethoxy)ethanol	0.358 (0.127)	0.446 (0.126)	0.315 (0.063)	0.457 (0.078)
unidentified 1*	1303 (359)	1476 (319)	2754 (826)	4405 (742)
2-ethyl-1-hexanol	0.316 (0.095)	0.547 (0.131)	0.208 (0.082)	0.478 (0.107)
5-isopropenyl-1-methyl-1-cyclohexene	4.228 (0.327)	5.359 (0.770)	0.002 (0.001)	0.023 (0.011)
acetophenone	0.106 (0.019)	0.164 (0.029)	0.058 (0.013)	0.103 (0.014)
2-nonanone	0.019 (0.005)	0.038 (0.012)	0.021 (0.008)	0.040 (0.007)
unidentified 2*	21 (22)	127 (52)	1889 (719)	2762 (621)
unidentified 3*	1134 (495)	1408 (438)	677 (222)	1348 (316)
2-decanone	0.019 (0.006)	0.038 (0.012)	0.029 (0.010)	0.065 (0.012)
2-isopropylphenol	0.043 (0.011)	0.047 (0.010)	0.030 (0.007)	0.053 (0.010)
benzothiazole	0.041 (0.009)	0.058 (0.012)	0.023 (0.005)	0.036 (0.006)
2-undecanone	0.008 (0.004)	0.015 (0.005)	0.009 (0.004)	0.024 (0.006)
1,3-diacetylbenzene	0.158 (0.034)	0.135 (0.022)	0.275 (0.051)	0.434 (0.064)
diethyl pthalate	0.084 (0.021)	0.067 (0.011)	0.047 (0.011)	0.103 (0.022)
1,3-diphenyl propane*	5747 (1864)	7613 (1951)	2301 (686)	7699 (2026)

*We could not determine concentrations of these compounds, and so mean single ion counts are presented for unidentified compounds 1 (m/z 110), 2 (m/z 124) and 3 (m/z 97), and 1,3-diphenyl propane (m/z 92), for which no authentic standard was commercially available.
